# Home Range and Habitat Selection of Blue-Eared Pheasants *Crossoptilon auritum* During Breeding Season in Mountains of Southwest China

**DOI:** 10.3390/ani15142015

**Published:** 2025-07-08

**Authors:** Jinglin Peng, Xiaotong Shang, Fan Fan, Yong Zheng, Lianjun Zhao, Sheng Li, Yang Liu, Li Zhang

**Affiliations:** 1 MOE Key Laboratory for Biodiversity Science and Ecological Engineering, Beijing Normal University, Beijing 100875, China; jinglin.peng@foxmail.com (J.P.); shangxt@foxmail.com (X.S.); 2 School of Ecology and State Key Laboratory of Biological Control, Sun Yat-sen University, Shenzhen 518107, China; liuy353@mail.sysu.edu.cn; 3 School of Life Sciences and Institute of Ecology, Peking University, Beijing 100871, China; fanf@szu.edu.cn (F.F.); shengli@pku.edu.cn (S.L.); 4 Wanglang National Nature Reserve, Mianyang 622563, China; 13408169171@163.com (Y.Z.); wlzlj@sina.com (L.Z.)

**Keywords:** blue-eared pheasant, satellite tracking, Kernel Density Estimation, Hidden Markov Model, activity rhythm, movement ecology

## Abstract

The blue-eared pheasant (*Crossoptilon auritum*), a Near Threatened (NT) species endemic to China, is primarily distributed across the northeastern region of the Qinghai–Tibetan Plateau. To bridge the fine-scale spatiotemporal gap in blue-eared pheasant behavioral ecology, this study combines satellite telemetry, movement modeling, and field-based habitat assessments (vegetation, topography, human disturbance). This multidisciplinary approach reveals detailed patterns of their behavior throughout the breeding season. Using satellite-tracking data from six individuals (five males tracked at 4 h intervals; one female tracked hourly) in Wanglang National Nature Reserve (WLNNR), Sichuan Province during breeding seasons 2018–2019, we quantified their home ranges via Kernel Density Estimation (KDE) and examined the female movement patterns using a Hidden Markov Model (HMM). The results indicated male core (50% KDE: 21.93 ± 16.54 ha) and total (95% KDE: 158.30 ± 109.30 ha) home ranges, with spatial overlap among individuals but no significant temporal variation in home range size. Habitat selection analysis indicated that the blue-eared pheasants favored shrub-dominated areas at higher elevations (steep southeast-facing slopes), regions distant from human disturbance, and with abundant animal trails. We found that their movement patterns differed between sexes: the males exhibited higher daytime activity yet slower movement speeds, while the female remained predominantly near nests, making brief excursions before returning promptly. These results enhance our understanding of the movement ecology of blue-eared pheasants by revealing fine-scale breeding-season behaviors and habitat preferences through satellite-tracking. Such detailed insights provide an essential foundation for developing targeted conservation strategies, particularly regarding effective habitat management and zoning of human activities within the species’ range.

## Simple Summary

The blue-eared pheasant (*Crossoptilon auritum*), a Near Threatened (NT) species endemic to China, is distributed across the northeastern region of the Qinghai–Tibetan Plateau. This study integrates satellite telemetry, movement modeling, and field-based habitat assessments (vegetation, topography, human disturbance). This multidisciplinary approach reveals detailed behavioral patterns throughout the breeding season. Using satellite-tracking data from six individuals in Wanglang National Nature Reserve (WLNNR), Sichuan Province, during the 2018–2019 breeding seasons, we quantified their home ranges and examined the female movement patterns. The results indicated that male core (50% KDE: 21.93 ± 16.54 ha) and total (95% KDE: 158.30 ± 109.30 ha) home ranges, showed spatial overlap among individuals but no significant temporal variation in home range size. Habitat selection analysis indicated that the blue-eared pheasants favored shrub-dominated areas at higher elevations (on steep southeast-facing slopes), regions distant from human disturbance, featuring abundant animal trails. We found that movement patterns differed between sexes: males exhibited higher daytime activity yet slower movement speeds, while females remained predominantly near nests, making brief excursions before returning promptly. These results reveal fine-scale behavioral patterns during the breeding-season and habitat preferences identified using satellite-tracking and provide an essential foundation for developing targeted conservation strategies.

## 1. Introduction

The home range of populations of different species of animals varies greatly [1,2]. It is influenced by various factors, including animal size, sex, diet, competition, and predators. Identifying the influence of these factors allows us to determine the limits of variation of the home range of species. The Phasianidae family (Galliformes), comprising ground-dwelling birds such as pheasants, exhibits distinctive morphological adaptations—including cranial crests, short beaks, limited flight capacity, and robust locomotor anatomy—that reflect their terrestrial lifestyle [3]. Within this family, all four Crossoptilon species—endemic to China—are classified as Least Concern (LC) by the IUCN, but only blue-eared pheasants (*Crossoptilon auritum*) are decreasing. However, under China’s Red List of Vertebrates, the brown eared-pheasant (*Crossoptilon mantchuricum*) is listed as Vulnerable (VU), while the white-eared pheasants (*Crossoptilon crossoptilon*), Tibetan-eared pheasants (*Crossoptilon harmani*), and blue-eared pheasants are Near Threatened (NT) [4]. All four are nationally protected, reflecting their ecological and evolutionary significance.

Notably, the blue-eared pheasant—a forest specialist with a broader distribution than its congener *C. mantchuricum*—serves as an ideal model for addressing critical knowledge gaps in the behavioral ecology of the understudied forest-dwelling *Crossoptilon* clade. Investigating its breeding-season ecology is pivotal for informing conservation strategies for this phylogenetically distinct group. Meanwhile, distributed across Qinghai, Gansu, Sichuan, and Ningxia, the blue-eared pheasant occupies a biogeographical crossroads at the interface of the Qinghai–Tibet Plateau, Southwest China, North China, and Mengxin regions [4]. Its range encompasses ecologically complex montane forests along the eastern Qinghai–Tibet Plateau, Qilian, Helan, and Hengduan mountain systems. These habitats, however, are increasingly threatened by anthropogenic pressures and climate change, necessitating urgent research to elucidate species-specific adaptations and vulnerabilities.

Reproductive challenges further imperil *Crossoptilon* populations. Low survival rates of eggs and chicks, exacerbated by parasitic infections (e.g., fowl typhoid) and nest predation, compound their inherent sensitivity to disturbance [5]. Such pressures underscore the need for targeted conservation interventions to preserve these endemic species, which represent irreplaceable evolutionary lineages and play keystone roles in ecosystem stability. For instance, pheasants contribute to seed dispersal, litter decomposition, and trophic dynamics by serving as prey for mesopredators and corvids. Population declines in these pheasants often signal broader ecosystem degradation, making their conservation a proxy for montane forest health.

Despite their ecological importance, previous studies on *C. auritum* and its sister species primarily rely on conventional methods with limited spatiotemporal resolution—direct observations, camera-trapping, and indirect sign surveys (e.g., droppings, molted feathers, feeding signs)—to infer their distributions and habitat use [6,7]. While species distribution models (e.g., MaxEnt) have been applied, trace-based data lack individual-level resolution, obscuring fine-scale movement patterns, intra-population variability, and behavioral plasticity. Satellite-based tracking and telemetry offer a transformative alternative, enabling high-frequency tracking of individual movement paths, home ranges, and habitat selection—critical for validating and refining existing ecological models [8,9,10].

The reproductive ecology of the blue pheasant exhibits a clear sexual division of labor. The breeding season varies by region but typically occurs from late April to early June, with clutch sizes ranging from 6 to 12 eggs [11]. The eggs are light turquoise or light brown, often with slightly yellowish speckles [12]. The incubation period lasts around 26 days, during which the female is responsible for incubating the eggs while the male remains nearby, keeping watch and foraging around the nest. After pairing, the male and female stay together, with the male frequently guarding the female as she forages. The chicks’ diet primarily consists of tender shoots, leaves, flower buds, and insects, with the exact composition varying seasonally. During the breeding period, males and females exhibit distinct behavioral patterns and movement traits. Notably, the male demonstrates strong protective behavior, while the female shows a pronounced attachment to the nest—roles that are particularly evident in their coordinated efforts at protection and foraging [11].

In this study, we hypothesize that during the breeding season: (1) blue-eared pheasants (BEP will be referred to in the following) in resource-rich areas will maintain more compact core ranges, potentially leading to home-range overlap; (2) males and females will display distinct movement patterns; (3) individuals will preferentially select microhabitats that minimize human disturbance while remaining close to critical resources. To test these predictions, we quantify core-range dynamics using Kernel Density Estimation (KDE), characterize sex-specific activity states with Hidden Markov Models (HMMs), and evaluate fine-scale environmental variables to assess habitat preferences. Our study provides detailed baseline data on the breeding-season behavioral ecology of China’s endemic BEP and other forest-dwelling pheasants, laying a critical foundation for their conservation in an era of rapid environmental change.

## 2. Materials and Methods

### 2.1. Study Area

Wanglang National Nature Reserve (WLNNR; 32°49′–33°02′ N, 103°55′–104°10′ E) was established in 1965 as one of China’s first giant panda (*Ailuropoda melanoleuca*) reserves in Sichuan Province. Located at the northern edge of the Hengduan Mountains, the 323 km^2^ reserve lies at the ecotone between the Sichuan Basin and the Qinghai–Tibetan Plateau, characterized by the rugged terrain and broad elevation range from 2300 to 4980 m (mean: 3200 m). The typical sub-alpine climate is cool and semi-humid, with a mean annual temperature of 2.5–2.7 °C (January: −6.1 °C; July: 12.7 °C) and precipitation of 859.9 mm [13,14,15]. Located within the global biodiversity hotspot, in the Mountains of Southwest China, WLNNR harbors a rich and unique fauna, including 11 pheasant species and some iconic large mammals such as giant pandas, golden snub-nosed monkeys (*Rhinopithecus roxellana*), and snow leopards (*Panthera uncia*) [6,16,17]. However, livestock grazing activities have been emerging as a critical disturbance within the reserve, posing substantial threats to the wildlife species inhabiting the forests of WLNNR [6,18,19].

### 2.2. Satellite-Tracking Dataset

In 2018, we established 10 pitfall traps each at Jincaopo and Muyangchang, with traps spaced approximately 5 m apart. These two sites, situated about 11 km apart within WLNNR, were selected due to frequent BEP activity. However, due to a mountain flood during the rainy season in 2018, the mobile signal was disrupted at Jincaopo, restricting trapping efforts in 2019 solely to Muyangchang. Traps were fitted with alarms and checked promptly 1–6 times daily throughout both trapping seasons. Captures occurred randomly concerning sex. However, the rugged mountainous terrain and the need to capture birds without causing injury introduced biases in both sample size and sex ratio. In the end, we were only able to track five males and one female. Despite these limitations, the detailed capture data provide valuable baseline information—though they do not imply that all individuals of the species exhibit the same behaviors. From April to May 2018 and 2019, we captured six BEPs (*C. auritum*) in WLNNR using custom-assembled foot-arch traps [20]. Five males and one female were successfully fitted with satellite-tracking loggers: males carried YH-GTCG0330 devices (Hangzhou Yuehai Technology Co., Ltd., Hangzhou, China) programmed to record locations at 4 h intervals (4 fixes per cycle), transmitting data via mobile networks before entering a 1 h dormancy phase. The female was equipped with an HQBG2715P logger (Hunan Global Messenger Technology Co., Ltd., Changsha, China) set to log hourly positions (5 fixes per cycle), with data transmission followed by a sleep period until the next cycle commenced.

### 2.3. Vegetation Survey

We delineated pheasant distribution ranges using 2018–2019 tracking data in ArcGIS 10.8 (ESRI). We defined our survey area based on existing tracking data of BEPs. After excluding outliers, we determined a 95% home-range boundary using the Minimum Convex Polygon (MCP) method. Within this boundary, vegetation sampling of 59 quadrats (10 m × 10 m) was systematically established at 30 m intervals in Jincaopo and Muyangchang: 32 in Muyangchang and 27 in Jincaopo. Plots were centered on Lat/Lon coordinates, except those near cliffs (<5% of total), which were adjusted by 5–10 m to ensure accessibility and safety. Field observations were conducted, and 20 ecological factors of these plots were recorded—altitude, slope, aspect, slope position, distance to road, salt feeding sites or water sources, width of animal trails, number of animal trails and dung piles, habitat type, horizontal openness, canopy density, tree species and mean tree diameter at breast height, shrub species and mean shrub diameter at breast height, mean shrub height, herbaceous cover, and moss cover.

### 2.4. Data Analysis

#### 2.4.1. Home Range Estimation

Kernel Density Estimation (KDE) was employed to quantify home ranges across study areas, with 95% and 50% probability contours representing total and core activity areas, respectively. Prior to analysis, satellite-tracking data were filtered to exclude outliers (e.g., erroneous signals or points outside reserve boundaries). Home ranges were calculated using the Home Range Tool 2.0 (HRT 2.0; ArcGIS 10.8, ESRI) and validated via the ctmm package (v1.2.0) in R (v4.3.3) to account for autocorrelation in movement data [21]. Overlap between individuals was assessed using the Overlap Index (OI) [22], while interindividual variability in home range size, spatial overlap, and potential resource sharing were analyzed using ctmm. Since there was only one set of female data, and she was followed for a short period, no overlap comparison was made. Temporal effects on home range size were evaluated via single-factor repeated-measures ANOVA.

#### 2.4.2. Habitat and Resource Selection Functions

Eighteen ecological variables were assessed for normality using the Kolmogorov–Smirnov test. Non-numeric factors (e.g., aspect, slope position, distance to roads/salt licks/water) were categorized and assigned ordinal values (Table S3). Spearman’s rank correlation analysis was employed to assess collinearity. Chi-square tests evaluated associations between habitat type, dominant tree species, slope aspect, and slope position. Normally distributed variables (*p* > 0.05) were analyzed using two-sample t-tests; non-normal variables were assessed via Mann–Whitney U tests [23]. Resource Selection Functions (RSFs) were modeled as [24]:

ω (x) = exp (β_0_ + β_1_x_1_ + β_2_x_2_+…+β_k_x_k_) (1)

where x denotes habitat covariates, and β_k_ represents selection coefficients estimated via logistic regression. Selection probabilities T(x) were derived as:

(2) $$T(x)=\frac{exp(\beta_{0}+\beta_{1}x_{1}+\beta_{2}x_{2}+\ldots +\beta_{k}x_{k})}{1+exp(\beta_{0}+\beta_{1}x_{1}+\beta_{2}x_{2}+\ldots +\beta_{k}x_{k})}$$

constrained to [0, 1], with T(x) = 1 indicating high utilization. Model performance was validated using ROC curves (SPSSAU v24.0).

#### 2.4.3. Behavioral States and Activity Rhythms

Given the coarse temporal resolution of male satellite-tracking data (4 h intervals), Hidden Markov Models (HMMs) were applied solely to the female’s high-frequency data (1 h intervals) to infer behavioral states. Using the moveHMM package (1.10) in R, step lengths and turning angles were derived from geocoordinates. Based on device accuracy and common sense, three states were defined: resting (short steps, low directional persistence), slow movement (intermediate steps), and transit (long, directional steps). The model was fitted to the data, generating state-dependent trajectories and probability graphs for the breeding season.

## 3. Results

### 3.1. Home Range Dynamics

**Figure 1:**
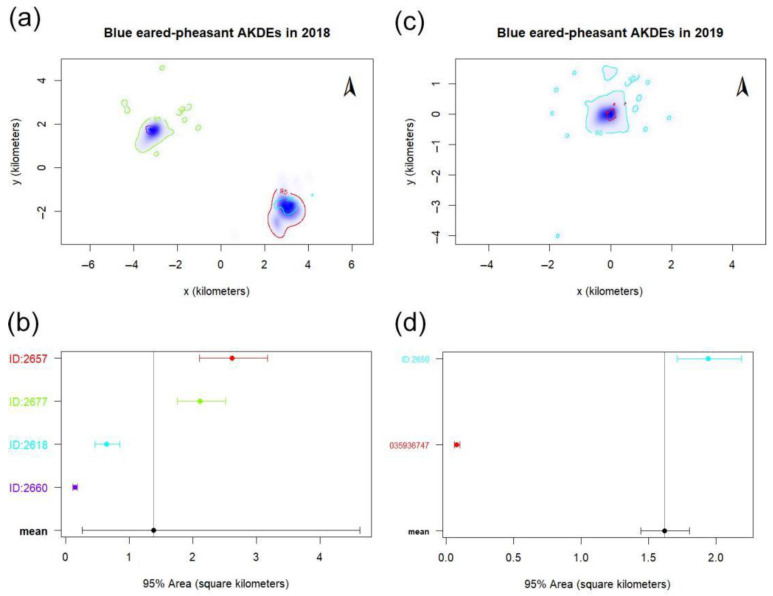
Home range estimates of blue-eared pheasant (*Crossoptilon auritum*) derived via continuous-time movement modeling (ctmm). (**a**) Utilization distribution of satellite-tracked individuals in 2018; (**b**) 95% Kernel Density Estimation (KDE) home range boundaries for 2018. (**c**) Utilization distribution in 2019; (**d**) 95% KDE home range boundaries for 2019. The meaning of the color corresponds to the device ID number.

The individuals tracked in this study were located between 2570 and 3635 m, aligning with previous findings. Home range sizes of BEPs in WLNNR varied significantly from 10.01 to 253.33 ha (HRT 2.0; Table 1, Figure 1 and Figure S1). Estimates derived from continuous-time movement modeling (ctmm) corroborated these findings. Male home ranges were consistently larger than the females’ (mean male: 158.30 ± 109.30 ha; female: 21.93 ha). One male (ID: 2618 recaptured as 2650 in 2019) demonstrated interannual site fidelity. Single-factor repeated-measures ANOVA (SPSS v22.0.0.0) revealed no significant temporal effect on home range size, regardless of whether late breeding-season (July–August) or early-season (May) data were excluded (Table S1). Overlap Index (OI) analysis (95% KDE) indicated moderate spatial overlap among males (mean OI: 0.48; Table 2). Female overlap was not assessed due to limited tracking duration. Cross-validation using ctmm confirmed overlapping home ranges among contemporaneous individuals (Table S2; Figure S2).

Due to the large differences in the tracking date and days of each individual, the relationship between home range size and time of blue eared-pheasant was analyzed by one-way repeated measurement ANOVA (Table S1), using SPSS version 22.0.0.0. The results showed that there was no significant difference in the area of the calculated home range sizes by excluding the site data in July and August near the end of the breeding period, or only taking the tracking site in May.

Based on 95%KDE home range, the Overlap Index (OI) was used as the standard to compare the overlap degree of home range between two blue-eared pheasants (Table 2). The average OI is 0.48 between males, and the OI is 0.54 between the same individual’s home range in two years.

### 3.2. Habitat Selection During the Breeding Season

#### 3.2.1. Habitat Characteristics

In Jincaopo, the arboreal layer is dominated by *Abies faxoniana* and *Betula albosinensis*. The shrub layer is characterized by dominant species such as *Spiraea schneideriana* and *Lonicera* spp., with a high coverage of herbaceous plants and mosses. Scattered bamboo are occasionally observed. In Muyangchang, the arboreal layer is primarily composed of *Picea purpurea*, *Abies faxoniana,* and *Betula albosinensis*. The shrub layer is dominated by *Salix spp.* and *Rosa omeiensis*. Herbaceous and moss cover is relatively low, with occasional sightings of moderately dense, mixed bamboo stands. From 59 plots (23 high-utilization, 36 low-utilization), key environmental features included steep slopes (<50°), high canopy density (>0.8), mid-to-lower slope positions, and remoteness from roads and salt licks (Figure S3).

#### 3.2.2. Ecological Drivers of Habitat Use

**Figure 2:**
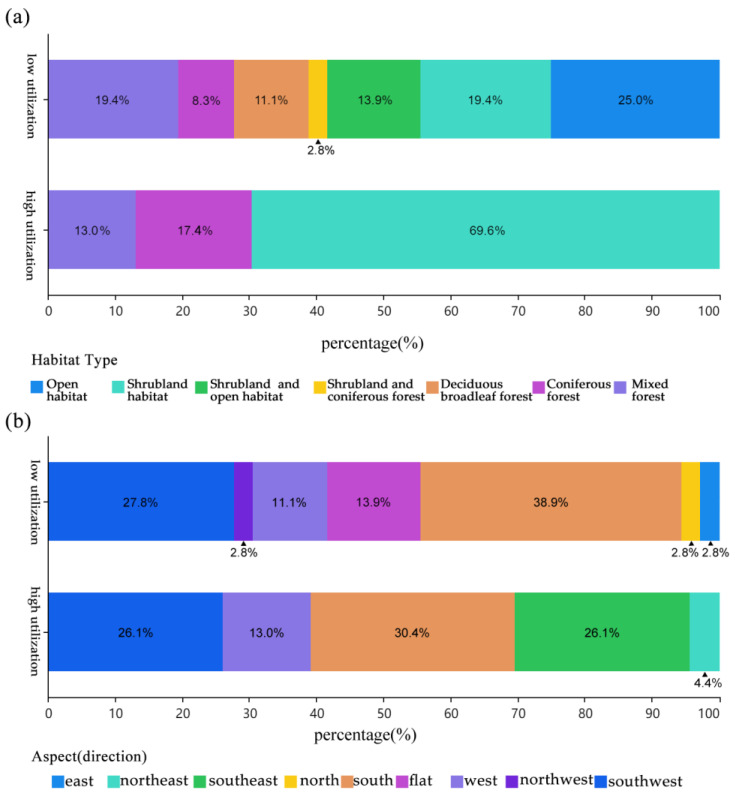
Habitat selection patterns of blue-eared pheasants (*Crossoptilon auritum*) in Wanglang National Nature Reserve (WLNNR): Chi-square contingency table analysis of utilization intensity (high vs. low) across (**a**) habitat types and (**b**) slope aspects.

Kolmogorov–Smirnov tests identified horizontal openness, mean shrub height, and herbaceous cover as normally distributed (*p* > 0.05); all other variables (*n* = 15) deviated from normality (*p* < 0.05; Table S4). Chi-square tests revealed significant associations between utilization intensity and slope aspect (χ^2^ = 12.7, *p* < 0.01) and habitat type (χ^2^ = 9.3, *p* < 0.05), with preference for southeast-facing slopes and shrub habitats (Table S6; Figure 2). T-tests and Mann–Whitney U tests further distinguished high-utilization habitats by steeper slopes (*p* < 0.05), greater distance to salt licks (*p* < 0.05), and higher animal trail density (*p* < 0.01; Table S5; Table S7).

#### 3.2.3. Resource Selection Model

Given that most ecological variables deviated from normality (Kolmogorov–Smirnov test, *p* < 0.05). Significant correlations (correlation coefficient B > 0.5) were identified between the following pairs: altitude and slope, distance to salt licks and water sources, canopy density and shrub metrics (mean shrub diameter and height), and mean tree diameter and herbaceous cover. Variables with non-significant correlations (correlation coefficient B < 0.5) were retained, while six collinear predictors—slope, distance to salt licks, distance to water sources, mean shrub diameter, mean shrub height, and mean tree diameter—were excluded to mitigate multicollinearity in subsequent analyses. A binary logistic regression incorporating the remaining variables revealed altitude, slope aspect, and animal trail density as significant predictors of habitat selection (*p* < 0.05; Table S8).

The resource selection function model, formulated with the chosen ecological factor variables, is represented as:

(3) $$T\left(x\right)=P=\frac{e^{z}}{1+e^{z}}$$

where

(4) $$\begin{array}{ll} z=\beta_{0}+\beta_{1}x_{1}+ & \beta_{2}x_{2}+\ldots +\beta_{k}x_{k}=ln(\frac{p}{1-p})\\ & =-33.087+0.012\times altitude-0.650\\ & *\;slope\;aspect\;(direction)\;+0.849\;*\;slope\;position-0.537\\ & *\;distance\;to\;road+0.006\;*\;mean\;width\;value+1.215\\ & *\;total\;number\;of\;animal\;trails-0.168\\ & *\;number\;of\;dung\;piles-2.849\\ & *\;mean\;horizontal\;openness+0.824\\ & *\;mean\;canopy\;density-0.052*\;mean\;shrub\;height\\ & -0.006*\;mean\;herbaceous\;cover+0.027\\ & *\;mean\;moss\;cover \end{array}$$

**Figure 3:**
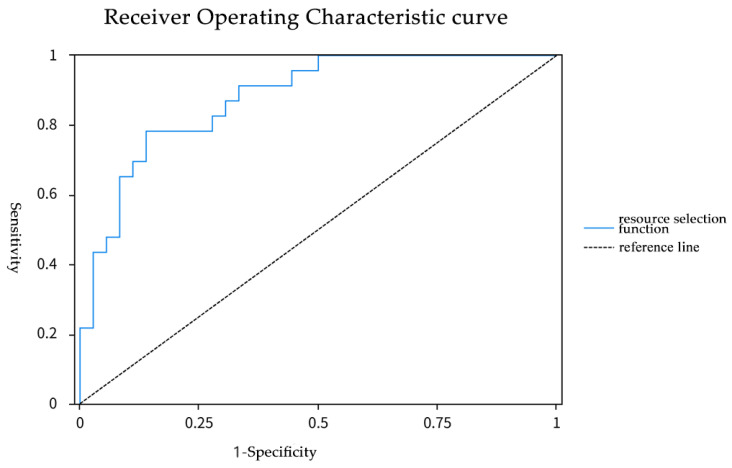
ROC curve of resource selection function model. The closer the area under the ROC curve (AUC) is to 1, the more reliable the model. In this study, the AUC was 0.879, which exceeds 0.5 and falls within the range of 0.7 to 0.9, indicating a high level of diagnostic accuracy.

Here, e represents the natural logarithm base, while P represents the probability of habitat selection. Model validation via ROC curve demonstrated strong accuracy (AUC = 0.879; Table S9, Figure 3).

### 3.3. Female Activity Patterns

#### 3.3.1. Movement Metrics

**Figure 4:**
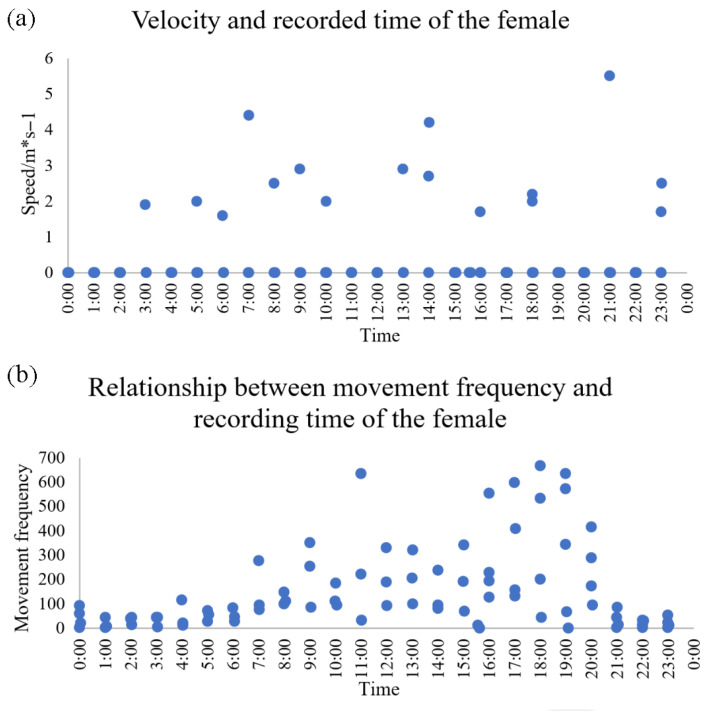
Correlation between movement speed and recording time for female blue-eared pheasants in scatter plot (**a**), and the relationship between movement frequency and recording time in scatter plot (**b**).

Male activity rhythms were not analyzed due to coarse satellite-tracker resolution (4 h intervals). The female exhibited sustained mobility, with movement speeds > 1 km/h during non-stationary periods (Figure 4a). Peak activity occurred between 15:00 and 19:00, with frequent movements from 5:00 to 15:00 (Figure 4b).

#### 3.3.2. Behavioral States Analysis via HMM

**Figure 5:**
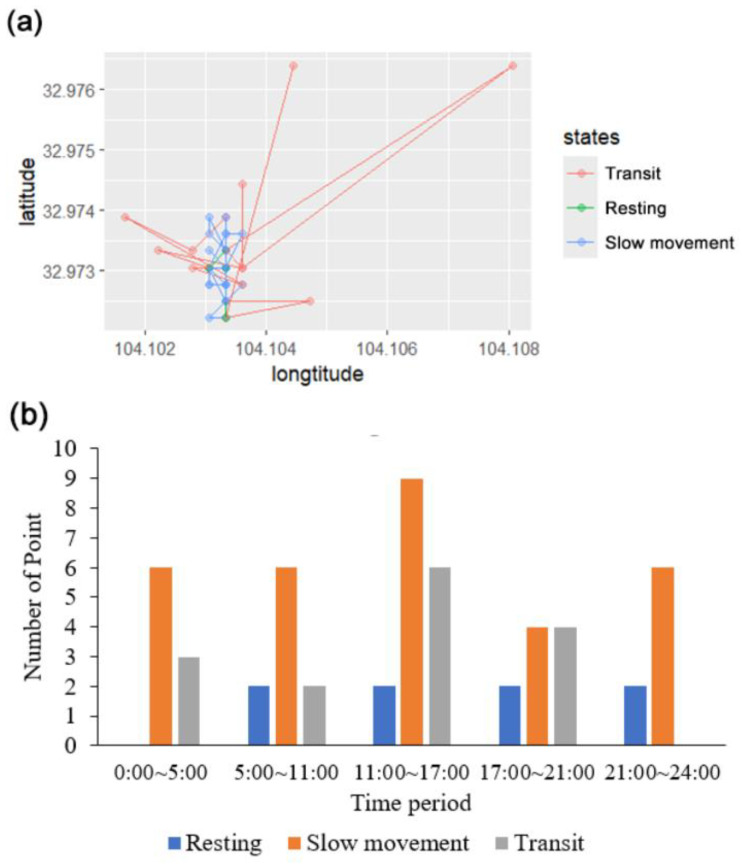
(**a**) Movement trajectory map of the female blue-eared pheasant. (**b**) Frequency of each movement state across different time periods. Rapid movement was more frequent from noon to afternoon (11:00–17:00) and peaked in the evening (17:00–21:00). No rapid movement was observed between 21:00 and 24:00; however, some rapid movement occurred between 00:00 and 05:00.

Analysis of 55 points of the female’s satellite-tracker identified three states: resting (short steps, step length less than 30 m, high turning angles), slow movement, and transit (directional, rapid movement, step length more than 100 m). The female predominantly occupied slow movement (68% of fixes), with resting concentrated near 104.1035° E, 32.973° N (Figure 5a). Transit occurred during dispersal from this core area. Temporal partitioning revealed consistent resting rates but variable transit frequency across diurnal periods (Figure 5b).

## 4. Discussion

### 4.1. Home Range

During the breeding season in WLNNR, male BEPs exhibited an average core home range of 21.93 ± 16.54 ha and a total home range of 158.30 ± 109.30 ha. In contrast, the female—due to a shorter tracking period—had a smaller core home range of 1.18 ha and a total home range of 10.01 ha. The sd of the home-range data was relatively large because of the small size of the data and the large variation in follow-up time.

According to Li et al. (1985) [12], BEPs form flocks during the non-breeding season, which begin to disperse in late March. By early April, most individuals are observed in pairs. May marks the peak of the egg-laying season, during which females demonstrate strong nest fidelity, and males frequently remain nearby. The incubation period typically lasts around 20 days. Similarly, Zheng et al. (1983) [11] reported that females rarely leave the nest during incubation (26 days), while males stay in close proximity. Our study failed to validate this process due to data size limitations. But the overall pattern of activity is consistent.

Our tracking data from 12 May to 15 May 2019 showed that a monitored female remained consistently at the same location, likely indicating incubation behavior. Additionally, one male (device ID 2650), tracked in both 2018 and 2019, displayed substantial differences in home range size across the two years. Notably, in May 2019, the male’s home range expanded significantly with time. Between 18 May and 31 May 2019, his activity points were frequently located near a female, suggesting pair bonding during that breeding season.

In this study, overlapping home ranges were observed among male individuals occupying the same area. This suggests that territorial behavior among BEPs during the breeding season may be relatively weak. Notably, our data do not confirm whether males with highly overlapping home ranges were actively involved in pairing and reproduction during this breeding season. This overlap may indicate that certain areas contain more concentrated resources, attracting multiple individuals for habitat use.

### 4.2. Habitat Selection

BEPs in Wanglang National Nature Reserve (WLNNR) exhibited pronounced habitat preferences during the breeding season, selecting areas characterized by higher elevations (2570–3635 m), southeast-facing slopes, steeper gradients (>30°), and greater distances from anthropogenic disturbances such as salt licks (U = 320.5, *p* < 0.05; Table S5). These habitats also featured higher densities of animal trails, which likely enhance foraging efficiency and predator detection [7,11,21]. Such preferences align with the species’ ecological requirements for thermal regulation, nest concealment, and resource acquisition, as documented in analogous montane ecosystems [23,25].

Southeast-facing slopes, which receive prolonged solar exposure, were strongly preferred (χ^2^ = 12.7, *p* < 0.01; Figure 2), consistent with Li et al.’s 1985 [12] observation of nest sites on sunlit ridges. Shrublands and coniferous–broadleaf mixed forests were disproportionately utilized (>80% of high-use plots), providing dense understory cover critical for evading predators like leopard cats (*Prionailurus bengalensis*) [26]. In the Minshan area, birds—including pheasants and passerines—make up 10.5% of the total prey consumed by carnivores [26]. In contrast, open grasslands were avoided, reflecting a trade-off between foraging accessibility and predation risk [25]. This selectivity mirrors seasonal shifts reported in Gansu Province, where pheasants transition from forest floors to shrub-dominated habitats in summer to exploit invertebrate abundance [25].

In this study, Jincaopo was characterized by fragmented grasslands interspersed with scattered shrubs and bamboo. In this area, pheasants frequently occupied the lower slopes, where shrub cover provided moderate concealment. This suggests behavioral plasticity in balancing foraging opportunities and safety in suboptimal habitats. In Muyangchang, dense mid-slope shrublands near a reserve station were preferred despite their proximity to infrastructure, indicating a tolerance for low-intensity human activity. The reduced avoidance of roads in this area (*p* = 0.407; Table S5) contrasts with the stronger road avoidance typically observed in regions with higher traffic volumes [27].

The BEPs’ preference for southeast-facing slopes and shrub-dominated habitats likely enhances concealment from predators while offering thermal benefits and access to nutrient-rich food sources. Shrubland provides effective cover from both ground predators (e.g., ocelots, yellow-throated martens) and aerial predators (e.g., golden eagles, common buzzards). High-utilization plots (*n* = 23) exhibited steeper slopes (U = 273.0, *p* = 0.028), greater distances from salt licks (U = 320.5, *p* = 0.044), and higher animal trail densities (U = 184.0, *p* < 0.001) compared to low-utilization plots (n = 36). These findings align with Wu et al.’s [25] observations in Gansu Province, where breeding-season pheasants selected steep, elevated slopes—areas associated with increased arthropod biomass and reduced anthropogenic disturbance.

In WLNNR, steeper slopes in Jincaopo and Muyangchang supported dense shrub–tree complexes, whereas gentler lower slopes transitioned to open grasslands. Salt licks, intermittently provisioned by locals for free-ranging livestock, were avoided (*p* < 0.05), likely due to human activity associated with these sites. The resource selection function (RSF) model identified elevation, southeast aspect, and animal trail density as key predictors of habitat use (AUC = 0.879; Figure 3). Animal trails—correlated with grazing activity—likely facilitate movement through dense vegetation while providing access to dung-associated invertebrates [11]. Trail networks may also improve predator detection by enhancing visibility, a critical advantage for ground-nesting birds [7,21]. Paradoxically, although livestock grazing degrades habitats overall, livestock presence may indirectly benefit pheasants by deterring mesopredators (e.g., red foxes *Vulpes vulpes*) through frequent ungulate activity [27]. Post-grazing ban, monitoring trail persistence, and predator dynamics will clarify these trade-offs. Concurrently, community outreach programs should emphasize reducing illegal grazing and salt-lick placement near core habitats.

### 4.3. Activity Rhythms

In this study, BEPs exhibited predominantly diurnal activity patterns. During the breeding season, the female spent extended periods at the nest. This behavior enhances nest protection and foraging efficiency under ecological pressure. According to Zheng et al. (1983) [11], BEPs are active from 5:00 to 11:00 and 17:00 to 21:00 during summer, accumulating up to 12 h of daily activity. Our data indicate that BEPs exhibit consistent activity throughout the day, with activity generally concentrated between 7:00 and 20:00. Two distinct peaks occur at approximately 11:00 and 18:00, with a slight dip around midday (14:00). Post-oviposition, males tend to remain close to the nest. Here, satellite-tracking data indicated that male pheasants exhibited activity across all time intervals, potentially due to predator disturbances or satellite-tracker error. The recorded high-speed movement points—consistent with known flight speeds of 38–48 mph (and up to 60 mph when pursued) [28]—were considered within the biologically plausible range. Notably, more high-speed movements occurred after 5:00, aligning with previous findings. However, unlike past observations, no distinct midday lull or late-afternoon activity peak was evident. Due to the male satellite-tracker recording at 4 h intervals and lacking fine-scale movement frequency data, the temporal resolution was insufficient for detailed activity analysis.

Conversely, the satellite-tracker on the female pheasant recorded data hourly and included movement frequency, allowing a more precise assessment of activity rhythms. Her movements clustered around specific coordinates, likely indicating a nesting site, with occasional bursts of speed representing foraging trips. Male movement patterns—predominantly under 1 km/h—suggested nest guarding or foraging nearby during incubation. Prior studies have noted that males alternate brief foraging bouts with vigilance, while females typically forage in the afternoons for 30–40 min per session [11].

Satellite tracking offers insights, but limitations warrant consideration. Males were monitored at four-hour intervals—and the lone female even less—so fine-scale movements and behavioral shifts may have been missed. Focusing on select sites within Wanglang National Nature Reserve may not capture environmental heterogeneity or anthropogenic pressures (e.g., disturbance by dogs and livestock) across the species’ range. Concentrating on home-range size and habitat selection overlooks social interactions, reproductive success, and predator avoidance. Satellite telemetry is prone to signal loss and positional error. Moreover, infrared camera networks target large mammals, limiting pheasant monitoring. More motion-sensor cameras, higher-resolution tracking, and year-round studies are needed.

## 5. Conclusions

This study employed satellite-tracking to analyze the behavioral ecology of BEPs in Wanglang Nature Reserve (WLNNR) during breeding season, focusing on home range dynamics, habitat preferences, and activity patterns. Male pheasants exhibited an average core area of 21.93 ± 16.54 ha (50% KDE) and a home range of 158.30 ± 109.30 ha (95% KDE). Habitat selection analysis revealed strong preferences for high-elevation zones, southeast-facing slopes, steep gradients, and areas distant from human disturbances (e.g., salt licks). These habitats likely offer enhanced security and foraging opportunities. Notably, pheasants favored locations with abundant animal trails, which may facilitate efficient movement and resource access. Activity patterns were predominantly diurnal, with females displaying heightened nest fidelity during breeding. For conservation planning, priority should be given to protecting high-elevation, southeast-facing slopes and steep terrains with minimal anthropogenic disruption. Infrastructure development and livestock grazing in these critical habitats must be restricted. The affinity for animal trails underscores a nuanced interplay between grazing activities and pheasant ecology. While excessive grazing could degrade habitat quality, moderate trail presence may benefit pheasant movement and foraging. To safeguard blue-eared pheasants in Wanglang Nature Reserve, we recommend prioritizing the protection of high-elevation, southeast-facing slopes and steep gradient areas, which provide optimal thermal benefits, concealment, and abundant foraging opportunities. These core zones should be designated as no-take reserves, prohibiting infrastructure development and mechanized vehicles to preserve natural cover. Seasonal closures of grazing from May through August will further minimize disturbance during the breeding season. Grazing management must strike a careful balance: low-intensity, controlled stocking can sustain a network of animal trails that facilitate pheasant movement and resource access, whereas overgrazing must be avoided to prevent understory degradation. By coupling these spatial protections with adaptive grazing policies and active community engagement, we can maintain habitat integrity, support efficient movement and foraging, and ultimately ensure the long-term resilience of blue-eared pheasant populations in the face of mounting environmental pressures.

## Figures and Tables

**Table 1:** Breeding season home range metrics of blue-eared pheasants (*Crossoptilon auritum*): tracking duration, effective locations, and spatial extents.

Area	Sex	ID	Date of Tracking	Effective Tracking Time (Days)	Home Range Distribution Probability	Home Range Area (HRT ^2^ 2.0)/hm^2^	Home Range Area (ctmm ^2^)/hm^2^
Muyangchang	M ^1^	2618 ^3^	9 April 2018–20 April 2018	12	50%	11.42	67.34
					90%	49.32	
					95%	68.35	
Jincaopo	M	2660	22 April 2018–23 May 2018	32	50%	2.52	14.38
					90%	9.82	
					95%	14.59	
Muyangchang	M	2657	18 May 2018–13 August 2018	88	50%	46.17	258.19
					90%	171.41	
					95%	253.33	
Jincaopo	M	2677	25 May 2018–5 July 2018	42	50%	25.98	212.05
					90%	124.98	
					95%	241.75	
Muyangchang	M	2650 ^3^	18 May 2019–14 August 2019	89	50%	23.54	195.22
					90%	97.54	
					95%	213.49	
Muyangchang	F ^1^	IMEI:8613 59035936 747SN:HQP830	12 May 2019–15 May 2019	4	50%	1.18	7.87
					90%	7.00	
					95%	10.01	

Note: ^1^. M: Male; F: Female. ^2^. HRT: Home Range Tool; ctmm: Continuous-Time Movement Modeling. ^3^. Male ID 2618 was recaptured in 2019 as ID 2650, demonstrating interannual site fidelity.

**Table 2:** Spatial overlap metrics among male blue-eared pheasants (*Crossoptilon auritum*) in Wanglang National Nature Reserve during the breeding season. Values represent pairwise home range overlap areas (ha; lower diagonal) and Overlap Indices (OI ^1^; upper diagonal).

ID (Sex ^3^, Year)	2618 (M, 2018)	2660 (M, 2018)	2657 (M, 2018)	2677 (M, 2018)	2650 ^2^ (Mx, 2019)
2618 (M, 2018)	/	0	0.49	0	0.54
2660 (M, 2018)	0	/	0	0.25	0
2657 (M, 2018)	64.64	0	/	0	0.64
2677 (M, 2018)	0	14.58	0	/	0
2650 ^2^ (M, 2019)	65.19	0	148.66	0	/

Note: ^1^. OI: Overlap Index, calculated as the ratio of overlapping area to the total home range area of both individuals. ^2^. Individual 2650 represents a recaptured male (originally ID 2618 in 2018), demonstrating interannual site fidelity. ^3^. Overlap comparisons exclude the female due to insufficient tracking duration.

## Data Availability

All data generated or analyzed during this study are included in this published article [and its Supplementary Information files].

## Supplementary Materials

The following supporting information can be downloaded at https://www.mdpi.com/article/10.3390/ani15142015/s1: Figure S1: Satellite-tracking to obtain the home ranges of the blue-eared pheasant (*Crossoptilon auritum*) across two study sites in WLNNR, Mianyang, Sichuan Province, China (2018–2019): Jincaopo (left) and Muyangchang (right). Figure S2: Annual variation in home range overlap of blue-eared pheasants (*Crossoptilon auritum*) in WLNNR, Mianyang, Sichuan Province, China: (a) spatial overlap patterns among individuals tracked in 2018; (b) spatial overlap patterns among individuals tracked in 2019. Figure S3: Environmental characteristics of blue-eared pheasant (*Crossoptilon auritum*) home ranges in Wanglang National Nature Reserve, Sichuan Province, China. (a) Slope gradient distribution across sampling plots. (b,c) Frequency distributions of horizontal openness (b) and canopy density (c) within sample plots. (d–g) Probability of habitat use based on slope position (d) and proximity to anthropogenic/natural features: roads (e), salt feeding sites (f), and water sources (g). Table S1: Test of within-subject effects. Table S2: Dyadic home range overlap (%) between blue-eared pheasants (*Crossoptilon auritum*) in the same year and region. Table S3: Categorical coding of ecological factors for habitat analysis. Table S4: Descriptive statistics and one-sample Kolmogorov–Smirnov (K-S) test results for environmental variables. Table S5: Results of Mann–Whitney U-tests comparing habitat variables between high- and low-utilization sites for blue-eared pheasants (*Crossoptilon auritum*) during the breeding season. Values represent median ranks. Table S6: Results of Chi-Square (χ^2^) tests for categorical habitat factors. Table S7: Results of two-sample t-tests comparing habitat variables between high- and low-utilization sites for blue-eared pheasants (*Crossoptilon auritum*) during the breeding season. Values represent mean ± SD. Table S8: Results of binary logistic regression modeling habitat use by blue-eared pheasants. Table S9: Receiver Operating Characteristic (ROC) analysis of the resource selection function model.
