# Supplementary material for: Home Range and Habitat Selection of Blue-Eared Pheasants Crossoptilon auritum During Breeding Season in Mountains of Southwest China
Source: Animals (Basel). 2025 Jul 8;15(14):2015. doi: 10.3390/ani15142015 (PMC12291958; doi:10.3390/ani15142015)
Supplement: Supplementary file 1 [file animals-15-02015-s001.zip › animals-3701933-supplementary.pdf]

Table S1. Test of within-subject effects

Source		Type III Sum of Squares	df	Mean Square	F	Sig.
Tracking Time	Sphericity Assumed	23890.341	2	11945.170	4.135	0.074
	Green-house–Geisser	23890.341	1.044	22873.505	4.135	0.131
	Huynh-Feldt	23890.341	1.114	21452.032	4.135	0.126
	Lower-bound	23890.341	1.000	23890.341	4.135	0.135

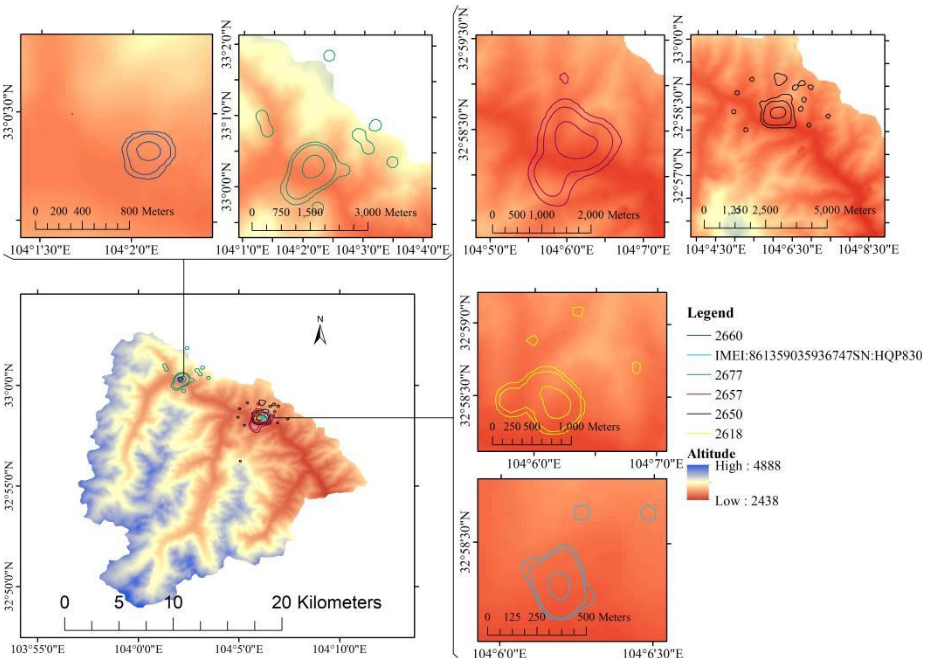

Figure S1. Satellite-tracking to obtain the home ranges of the blue-eared pheasant (*Crossoptilon auritum*) across two study sites in WLNNR, Mianyang, Sichuan Province, China (2018–2019): Jincaopo (left) and Muyangchang (right).

Table S2. Dyadic home range overlap (%) between blue-eared pheasants (*Crossoptilon auritum*) in the same year and region.

Individual A	Individual B	Percentage overlap/%
ID:2618	ID:2657	67.58
ID:2660	ID:2677	63.14
ID:2650	IMEI:861359035936747SN:HQP830	46.06

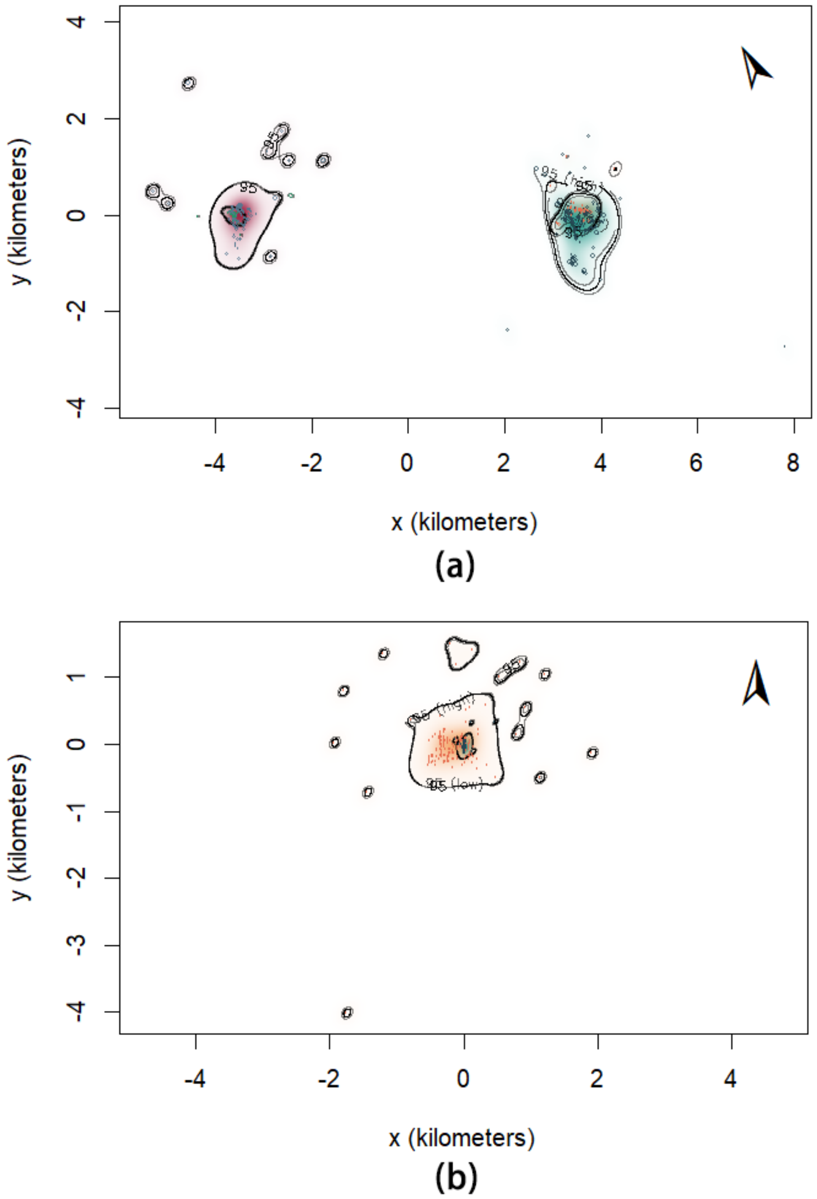

Figure S2. Annual variation in home range overlap of blue-eared pheasants (*Crossoptilon auritum*) in WLNNR, Mianyang, Sichuan Province, China: (a) spatial overlap patterns among individuals tracked in 2018; (b) spatial overlap patterns among individuals tracked in 2019.

Table S3. Categorical coding of ecological factors for habitat analysis.

Ecological Factor	Raw Data	Assignment
Aspect (direction)	East (67.5°~112.5°)	1
	Northeast (22.5°~67.5°)	2
	Southeast (112.5°~157.5°)	3
	North (337.5°~22.5°)	4
	South (157.5°~202.5°)	5
	Flat (-1)	6
	West (247.5°~292.5°)	7
	Northwest (292.5°~337.5°)	8
	Southwest (202.5°~247.5°)	9
Slope position	Upper slope	1

	Mid-slope	2
	Lower slope	3
	Flat	4
Distance to road/ salt feeding sites/ water sources	<10m	1
	10~50m	2
	>50m	3

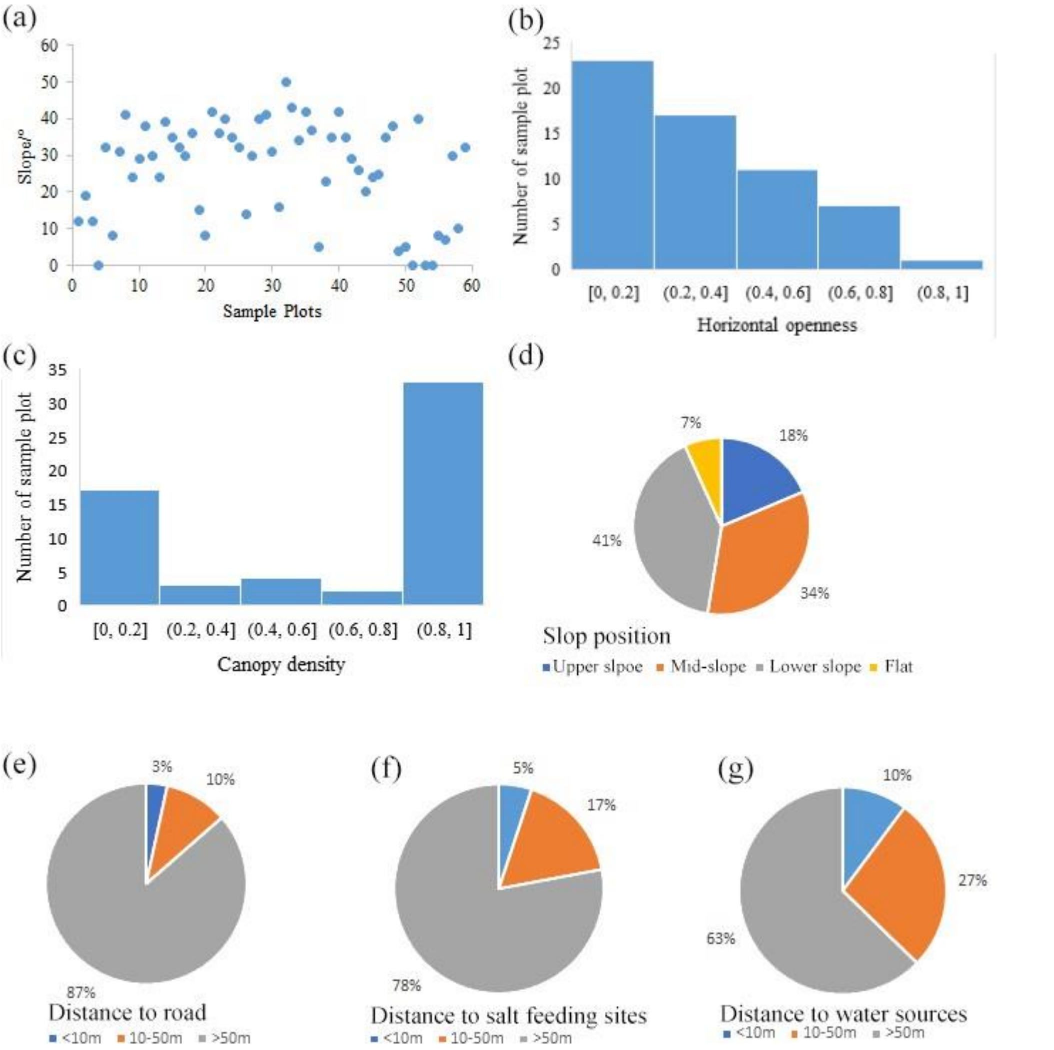

Figure S3. Environmental characteristics of blue-eared pheasant (*Crossoptilon auritum*) home ranges in Wanglang National Nature Reserve, Sichuan Province, China. (a) Slope gradient distribution across sampling plots. (b–c) Frequency distributions of horizontal openness (b) and canopy density (c) within sample plots. (d–g) Probability of habitat use based on slope position (d) and proximity to anthropogenic/natural features: roads (e), salt feeding sites (f), and water sources (g).

Table S4. Descriptive statistics and one-sample Kolmogorov–Smirnov (K-S) test results for environmental variables.

Factor	Mean	Standard deviation	Skew-ness	Kurtosis	Kolmogorov–Smirnov test	
					D-value	p
Altitude	2831.949	183.252	-0.022	-1.154	0.116	0.048*
Slope	25.949	13.593	-0.539	-0.893	0.165	0.000**
Aspect (degree)	180.373	75.761	-1.211	1.149	0.227	0.000**
Aspect (direction)	6.119	2.174	-0.053	-0.839	0.205	0.000**
Slope position	2.356	0.866	-0.114	-0.756	0.246	0.000**
Distance to road	2.763	0.625	-2.414	4.188	0.512	0.000**
Distance to salt feeding sites	2.610	0.766	-1.576	0.653	0.474	0.000**
Distance to water sources	3.017	1.333	-0.711	-1.408	0.397	0.000**
Width of animal trails	45.446	27.441	0.266	1.100	0.168	0.000**
Number of animal trails	2.729	1.760	-0.140	-0.965	0.171	0.000**
Number of dung piles	5.000	7.465	2.893	11.109	0.251	0.000**
Horizontal openness	0.313	0.218	0.684	0.024	0.093	0.233
Canopy density	0.621	0.394	-0.600	-1.461	0.256	0.000**
Mean tree diameter at breast height	8.333	12.218	1.035	-0.520	0.396	0.000**
Mean shrub diameter at breast height	4.396	4.340	1.886	4.957	0.156	0.001**
Mean shrub height	2.870	2.139	1.360	4.480	0.099	0.164
Herbaceous cover	67.818	18.585	-0.849	0.239	0.110	0.072
Moss cover	15.280	17.486	2.049	3.908	0.237	0.000**

* $p < 0.05$ ** $p < 0.01$.

Table S5. Results of Mann–Whitney U-tests comparing habitat variables between high- and low-utilization sites for blue-eared pheasants (*Crossoptilon auritum*) during the breeding season. Values represent median ranks.

Ecological Variable	Utilization $\bar{\chi}$		U	Z	p
	Low (n=36)	High (n=23)			
Altitude	2811.139	2864.522	350.000	-0.995	0.320
Slope	22.361	31.565	273.000	-2.194	0.028*
Aspect (degree)	174.028	109.304	402.000	-0.187	0.852
Aspect (direction)	6.417	5.652	325.000	-1.433	0.152
Slope position	2.333	2.391	406.500	-0.124	0.902
Distance to road	2.806	2.870	383.000	-0.811	0.407
Distance to salt feeding sites	2.611	2.913	320.500	-2.013	0.044*
Distance to water sources	2.444	2.652	373.000	-0.744	0.457
Width of animal trails	41.333	51.884	339.000	-1.169	0.243
Number of animal trails	2.056	3.783	184.000	-3.638	0.000**
Number of dung piles	6.194	3.130	336.500	-1.221	0.222
Canopy density	0.590	0.669	370.500	-0.676	0.499
Mean tree diameter at breast height	8.054	8.769	410.500	-0.064	0.949
Mean shrub diameter at breast height	4.344	4.496	351.000	-0.981	0.327
Moss cover	14.832	15.980	354.000	-0.933	0.351

Note: * $p<0.05$ ** $p<0.01$. Female excluded due to insufficient tracking duration (n = 1).

Table S6. Results of Chi-Square (χ^2) tests for categorical habitat factors.

Factor	χ^2	p
Aspect (direction)	16.408	0.037*
Slope position	4.188	0.242
Dominant tree species	10.531	0.650
Habitat type	22.492	0.001**

* $p < 0.05$; ** $p < 0.01$.

Table S7. Results of two-sample t-tests comparing habitat variables between high- and low-utilization sites for blue-eared pheasants (*Crossoptilon auritum*) during the breeding season. Values represent mean ± SD.

Factor	Utilization		t	p
	Low (n=36)	High (n=23)		
Horizontal openness	0.32±0.21	0.30±0.23	0.441	0.661
Mean shrub height	2.77±2.45	3.02±1.57	-0.424	0.673
Herbaceous cover	71.10±18.27	62.68±18.28	1.725	0.090

Note: * $p<0.05$; ** $p<0.01$. No variables showed statistically significant differences.

Table S8. Results of binary logistic regression modeling habitat use by blue-eared pheasants.

Variable	B	S.E.	z	Wald χ^2	Sig.	Exp(B)	95% C.I. for Exp(B)
Altitude	0.012	0.005	2.307	5.320	0.021	1.012	1.002~1.022
Aspect (direction)	-0.650	0.278	-2.340	5.477	0.019	0.522	0.303~0.900
Slope position	0.849	0.666	1.274	1.623	0.203	2.338	0.633~8.630
Distance to road	-0.537	1.173	-0.457	0.209	0.647	0.585	0.059~5.830
Width of animal trails	0.006	0.019	0.285	0.081	0.776	1.006	0.968~1.044
Number of animal trails	1.215	0.408	2.980	8.879	0.003	3.370	1.516~7.493
Number of dung piles	-0.168	0.122	-1.373	1.885	0.170	0.846	0.666~1.074
Horizontal openness	-2.849	2.144	-1.329	1.766	0.184	0.058	0.001~3.867
Canopy density	0.824	1.695	0.486	0.236	0.627	2.279	0.082~63.224
Mean shrub diameter at breast height	-0.052	0.283	-0.184	0.034	0.854	0.949	0.546~1.652
Herbaceous cover	-0.006	0.034	-0.180	0.032	0.857	0.994	0.930~1.063
Moss cover	0.027	0.026	1.045	1.091	0.296	1.028	0.976~1.082
Intercept	-33.087	14.517	-2.279	5.195	0.023	0.000	0.000~0.010

Table S9. Receiver Operating Characteristic (ROC) analysis of the resource selection function model.

Title	AUC	S.E.	Sig.	95% CI
resource selection function	0.876	0.044	0.000**	0.789~0.962

* $p < 0.05$; ** $p < 0.01$.
